# Identification of candidate genes linking systemic inflammation to atherosclerosis; results of a human *in vivo* LPS infusion study

**DOI:** 10.1186/1755-8794-4-64

**Published:** 2011-08-10

**Authors:** Suthesh Sivapalaratnam, Rosienne Farrugia, Max Nieuwdorp, Cordelia F Langford, Rachel T van Beem, Stephanie Maiwald, Jaap Jan Zwaginga, Arief Gusnanto, Nicholas A Watkins, Mieke D Trip, Willem H Ouwehand

**Affiliations:** 1 Department of Vascular Medicine, Academic Medical Center, Meibergdreef 15, 1105 AZ, Amsterdam, The Netherlands; 2 Department of Hematology, University of Cambridge, Long Road, CB2 0PT, Cambridge, UK; 3 Department of Human Genetics, Wellcome Trust Sanger Institute, Hinxton, CB10 1SA, Cambridge, UK; 4 Department of Experimental Immunohematology, Sanquin Research, Plesmanlaan 125, 1066 CX, Amsterdam, The Netherlands; 5 Department of Statistics, University of Leeds, Woodhouse lane, LS2 9JT, Leeds, UK; 6 National Health Service Blood and Transplant, Cambridge, Long Road, CB2 0PT, UK

**Keywords:** Human, Monocytes, LPS infusion, Transcriptome, In Vivo

## Abstract

**Background:**

It is widely accepted that atherosclerosis and inflammation are intimately linked. Monocytes play a key role in both of these processes and we hypothesized that activation of inflammatory pathways in monocytes would lead to, among others, proatherogenic changes in the monocyte transcriptome. Such differentially expressed genes in circulating monocytes would be strong candidates for further investigation in disease association studies.

**Methods:**

Endotoxin, lipopolysaccharide (LPS), or saline control was infused in healthy volunteers. Monocyte RNA was isolated, processed and hybridized to Hver 2.1.1 spotted cDNA microarrays. Differential expression of key genes was confirmed by RT-PCR and results were compared to *in vitro *data obtained by our group to identify candidate genes.

**Results:**

All subjects who received LPS experienced the anticipated clinical response indicating successful stimulation. One hour after LPS infusion, 11 genes were identified as being differentially expressed; 1 down regulated and 10 up regulated. Four hours after LPS infusion, 28 genes were identified as being differentially expressed; 3 being down regulated and 25 up regulated. No genes were significantly differentially expressed following saline infusion. Comparison with results obtained in *in vitro *experiments lead to the identification of 6 strong candidate genes (*BATF, BID, C3aR1, IL1RN, SEC61B *and *SLC43A3*)

**Conclusion:**

*In vivo *endotoxin exposure of healthy individuals resulted in the identification of several candidate gene*s *through which systemic inflammation links to atherosclerosis.

## Background

Inflammation and atherosclerosis are closely linked. In patients with chronic inflammation due to disorders such as rheumatoid arthritis or systemic lupus erythematodus (SLE), the incidence of cardiovascular disease (CVD) is 2 to 50-fold higher [1,2]. Even after correction for traditional risk factors, patients with chronic inflammatory disorders have accelerated plaque progression [3]. Circulating levels of hsCRP (high sensitive C-Reactive Protein), a biomarker for inflammation, are proven to be a strong, independent predictor of future myocardial infarction and stroke even among apparently healthy asymptomatic men [4]. Finally, in a cohort of healthy individuals, subjects with endotoxin levels beyond 50 pg/ml (90^th ^percentile) exhibited a threefold increased risk of cardiovascular events (OR [95% CI], 2.9 [1.4-6.3]; p < 0.01) [5].

*In vivo*, monocytes bridge inflammation and atherosclerosis. They express TLR4, the receptor for endotoxin and are involved in all key sequelae of atherosclerosis [5,6].

We hypothesized that endotoxin exposure *in vivo *results in changes in monocyte transcriptome that could lead to a more atherogenic phenotype. This would be reflected by differential expression of, among others, atherosclerosis related genes. We aimed to identify these atherosclerosis related genes as candidates for further investigation.

## Methods

### *In Vivo *Experiments

#### Endotoxin infusion in healthy volunteers

In order to mimic *in vivo *systemic inflammation associated with endotoxemia, we used a model in which healthy volunteers are exposed to lipopolysaccharide (LPS), the endotoxin derived from gram negative bacteria [7]. Informed consent was obtained from healthy Caucasian male volunteers for the study, which was approved by the Institutional Review Board of the Academic Medical Center Amsterdam. Inclusion criteria included: no history of sepsis or CVD; not having previously received endotoxin intravenously; non-smokers, no use of any medication and free from any febrile illness in the month preceding the study. In all subjects, a medical history, physical examination, routine laboratory examination, electrocardiogram and chest X-ray were performed. All experiments were performed after an overnight fast.

A bolus of *Escherichia coli *endotoxin (LPS; catalog number 1235503, lot G2B274; Pharmacopeial Convention, Inc, Rockville, USA; 1 ng/kg) was infused intravenously in healthy volunteers. For controls, an equal amount of endotoxin-free 0.9% NaCl (saline) was infused. Vital signs, including temperature of the study subjects, were monitored at the Intensive Care Unit by a medical doctor who was present throughout the experiments. The incidence, time and severity of clinical symptoms associated with endotoxemia, were recorded. Blood was regularly sampled for clinical chemistry and hematological parameters. IL-6 levels were determined using the Cytometric Bead Array technique (R&D systems, Minneapolis, MN, USA). Whole blood samples in 4% trisodium citrate were obtained at baseline (T = 0), one hour (T = 1) and four hours (T = 4) after LPS infusion.

#### Monocyte mRNA preparation

Whole blood was centrifuged 12 minutes at 300 g and plasma was replaced with an equal volume of PBS buffer containing 1.25% BSA and 2 mM EDTA (PBS/BSA/EDTA). The samples were then layered onto 0.5 volume Histopaque-1077 (Sigma-Aldrich, St. Louis, MO, USA) and centrifuged at 400 g for 20 minutes. Mononuclear cells were removed and washed twice with PBS/BSA/EDTA buffer. An aliquot of 1 million peripheral mononuclear blood cells were removed for flow cytometric analysis. The remaining cells were used for monocyte isolation using magnetic CD14+ microbeads (Miltenyi Biotech GMBH, Bergisch Gladbach, Germany) according to the manufacturer's instructions. Briefly, 20 μl microbeads and 80 μl PBS/BSA/EDTA containing 10^6 ^cells were mixed and incubated on ice for 15 minutes. Cells were washed with PBS/BSA/EDTA before being run on an MS column on a varioMACS (Miltenyi Biotech, Cologne, GMBH). Cell purity was assessed on an aliquot of cells by flow cytometry using anti-CD14 (CLB-mon/1, 8G3, Mouse IgG2a, Sanquin Reagents, Amsterdam, The Netherlands). The purified cells were then lysed in RNA Bee (TelTest Inc., Friendswood, Texas, USA) and RNA extracted according to manufacturer's protocol. The concentration of purified RNA was quantified using a NanoDrop spectrophotometer (NanoDrop Technologies, Wilmington, Delaware, USA) and quality assessed using an Agilent 2100 Bioanalyzer (Agilent, Palo Alto, CA, USA). RNA purity was assessed by Real-Time PCR (RT-PCR) using Taqman transcripts specific for B-cells (CD19/Hs00174333_m1), T-cells (CD3/Hs00167894_m1), erythroid cells (CD235a/Hs00266777_m1), platelets (CD41/HS00166246_m1), neutrophils (CD66a/Hs00174351_m1), monocytes (CD14/Hs00169122_g1) and GAPDH (Hs99999905_m1) as control. A total of 12 ng of RNA was reverse transcribed and amplified using Taqman Transcription Reagents according to the manufacturer's protocol (Applied Biosystems, Foster City, CA, USA). Assays were run on an Mx4000 Multiplex Quantitative PCR System (Stratagene Inc, La Jolla, California, USA). Only those samples with a FACS purity > 80% were processed to ensure reliability of results.

#### Monocyte cDNA preparation

Template Switching Polymerase Chain Reaction (TS-PCR) was used to prepare amplified double stranded (ds) copy DNA (cDNA) following manufacturer's instructions (BD Biosciences Clontech, Palo Alto, CA, USA). Amplifications were performed with either 10 ng (23 cycles) or 100 ng (17 cycles) of starting material, as described previously [8]. Amplified cDNA was subsequently purified using the QIAquick PCR Purification Kit (QIAGEN Inc., Chatsworth, UK) according to manufacturer's protocol with two amendments. Firstly, samples were washed three times in 0.7 mL PE buffer. Secondly, for elution, 50 μL of sterile water was added, the column was left to stand for 2 minutes and then centrifuged. Purified cDNA was quantified using a NanoDrop and fragment length assessed on 1.0% agarose gel. Performance of the amplification was assessed using RT-PCR as above.

#### Microarray processing

Out of the 11 LPS infusion experiments the 5 samples with the highest purity based on FACS and RT-PCR and sufficient yield were chosen for arraying at T = 0 vs. T = 1 and T = 0 vs. T = 4. Similarly, 4 out of the 5 were chosen for the control experiments for the T = 0 vs. T = 1 and 2 of the 5 samples for the T = 0 vs. T = 1. A total of 250 ng of amplified cDNA was labelled by incorporation of Cy3 or Cy5 dCTP using the Bioprime Labelling Kit (Invitrogen Ltd, Paisley, UK). Labelled products were purified on Autoseq G-50 columns (GE Healthcare, Buckinghamshire, United Kingdom) before differential labelling. Biologically paired pre- and post-infusion samples were pooled and hybridized to Hver 2.1.1 cDNA micro-arrays (Wellcome Trust Sanger Institute; WTSI). Hybridization and washing of the arrays were performed according to WTSI protocols. Processed slides were promptly scanned at 10- μm resolution on an Agilent Micro-array Scanner (G2505B; Agilent Technologies, Stockport, United Kingdom). Images were exported into GenePix version 4.1 (Molecular Devices, Sunnyvale, CA, USA) for spotfinding and feature extraction. In accordance with MIAME (Minimum Information About a Micro-array Experiments) regulations, all data were deposited into ArrayExpress database at http://www.ebi.ac.uk[9,10].

#### Data analysis

Data was analyzed using R-project version 2.2.0. Features were included if all of the following criteria were met: (i) Manually unflagged in Genepix 6.0 (Axon Instruments, Foster City, CA, USA); (ii) (Δ median-mean)/median intensity difference < 0.1;

(iii) Saturation < 20%; (iv) Mean/standard deviation (SD) > 2; (v) Intensities of background (BG) + 1SD > 90% and G + 2 SD > 80%. In addition, the feature had to be either up or down regulated in at least 50% of the arrays, in order to pass the inclusion threshold. LPS and control samples were analysed separately in a linear mixed effects model with corrections for false discovery rates (FDR) As cut-off we selected a FDR < 0.005, a posterior probability of differential expression > 0.95. The foldchanges had to be greater than two and present in at least 70% of the arrays.

#### Microarray results validation

RT-PCR was used to confirm the observations from the comparative microarray study in the 6 LPS samples not used for Microarray for the following transcripts chosen at random: SLA1 (Hs00153504_m1); BATF (Hs00232390_m1); C3aR1 (Hs00377780_m1); AKIP (Hs00610917_g1); ILRN (Hs00174099_m1); TIMP1 (Hs00171558_m1); VCAN (Hs00171642_m1).

RNA (100 ng) was reverse transcribed using the TaqMan RT kit (Applied Biosystems), from which 0.5 ng cDNA was then used as template for RT-PCR following the manufacturer's instructions (Applied Biosystems). Reactions were incubated at 50°C for 2 minutes then 95°C for 10 minutes, and RT-PCR reactions were performed over 40 cycles (95°C for 15 seconds, 60°C for 1 minute) on an MX-4000 (Stratagene, La Jolla, CA). Threshold values (Ct) were normalized to GAPDH to allow comparison between samples.

### *In Vivo *and *In Vitro *Data Synthesis

Datamining *in silico *of the differentially expressed genes was performed using public databases and tools including biomart, iHOP, pubmed and reactome. Data from the *in vitro *study was used to select genes which were differentially expressed in both the *in vivo *and *in vitro *experiments (see additional file 1, supplementary methods).

## Results

### *In Vivo *Experiments

#### Endotoxin infusion in healthy volunteers

**Figure 1:**
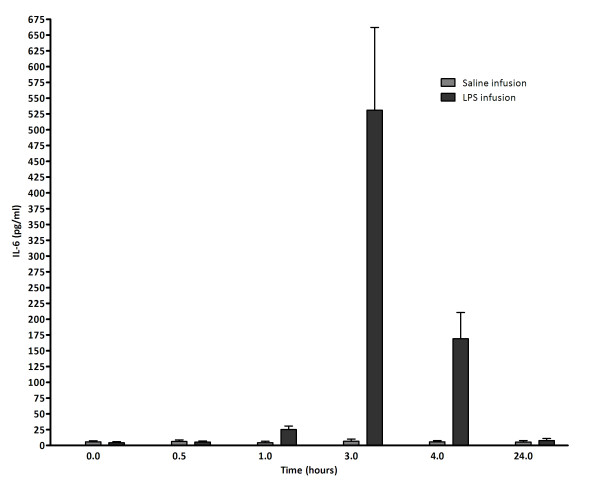
**IL-6 values increase to a maximum value three hours after LPS infusion**. IL-6 levels were determined using the Cytometric Bead Array technique (R&D systems, Minneapolis, MN, USA).

In total, 16 healthy men with an average age of 23 ± 1 years (mean ± SD) meeting the inclusion criteria participated; 11 for LPS infusion and five as controls. Medical history, physical examination, routine laboratory examination, electrocardiogram and chest X-ray were normal. All subjects receiving LPS experienced a clinical response consisting of nausea, malaise, chills, muscle ache and fever. Plasma/Serum IL-6 increased significantly (Figure 1). No such effects were observed in the control group.

#### Monocyte cDNA preparation

**Figure 2:**
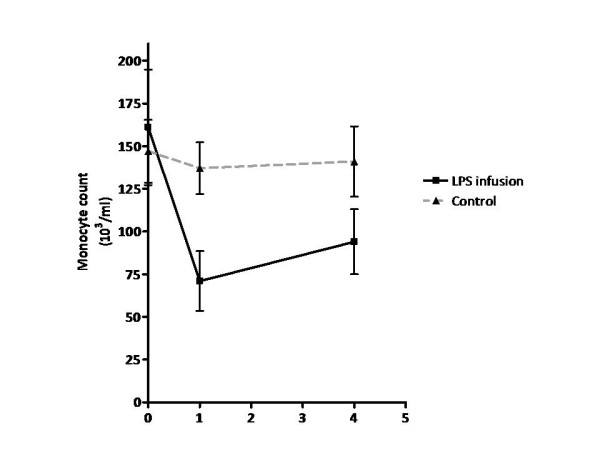
**Monocyte count is significantly lower in the LPS infusion experiments compared to saline at T = 1**.

The monocyte count differed significantly between LPS and control experiments at the first hour (p < 0.05) (Figure 2). In the LPS infusion experiments, monocyte count dropped 55% in the first hour followed by a slow increase towards normal at four hours. In the control experiments, monocyte count varied slightly but did not decrease as observed after LPS infusion (Figure 2). Summary data for all samples and those included in the study is shown in additional file 2 and 3; supplementary table 1 and 2. RT-PCR performed pre- and post-amplification demonstrated minimal contamination of selected RNA samples by other cell types and non-biased amplification (Data not shown).

#### Microarray processing

**Figure 3:**
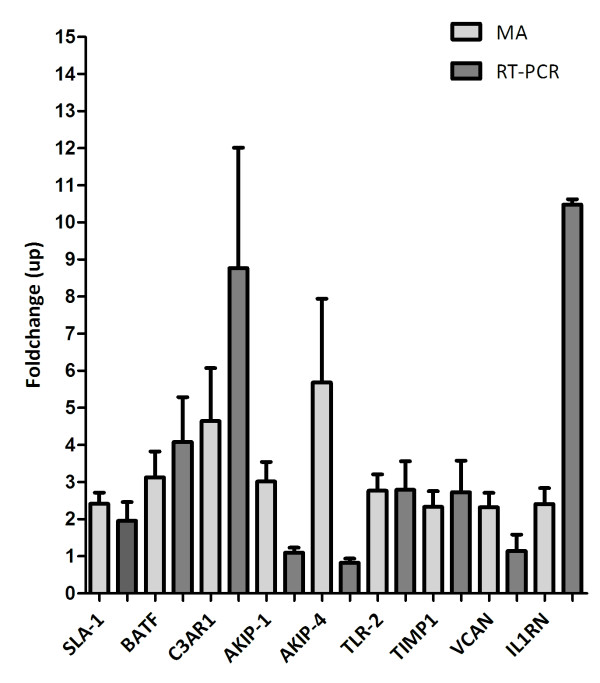
**Except for *AKIP*, microarray (MA) and RT-PCR fold changes are comparable**. *AKIP-1*; Differential expression of *AKIP *in one hour post infusion samples. *AKIP-4*; Differential expression of *AKIP *in four hours post infusion samples.

For the T = 0 vs. T = 1 experiments, 10 arrays were hybridised with LPS-stimulated samples and 8 with control samples. On average 19% of spots passed the stringent quality control criteria. One hour after LPS infusion (T = 1), 11 genes with a known function were differentially expressed, of which, 1 was down regulated and 10 unregulated (Table 1 and Figure 3) with fold-changes ranging from 2.0 to 5.8.

**Table 1:** Genes differentially expressed one hour after LPS infusion

Ensembl ID	HUGO geneID	Full name	foldchange	p-value
ENSG00000172409	CLP1	Pre-mRNA cleavage complex II protein Clp1	0,47	0,001
ENSG00000197766	CDF	Complement factor D precursor	2,02	0,003
ENSG00000175592	FOSL1	Fos-related antigen 1	2,05	0,001
ENSG00000175602	DIPA	Delta-interacting protein A	2,05	0,001
ENSG00000166008	MAGEA9	Melanoma-associated antigen 9	2,37	0,001
ENSG00000136689	IL1RN	Interleukin-1 receptor antagonist protein precursor	2,41	0,003
ENSG00000179271	PLINP-1	Papillomavirus L2-interacting nuclear protein 1	2,69	0,002
ENSG00000181667	PTPRCAP	Protein tyrosine phosphatase receptor type C-associated protein	2,85	0,005
ENSG00000175756	AKIP	Aurora kinase A-interacting protein	3,01	0,001
ENSG00000196783	CCL3L1	Small inducible cytokine A3-like 1 precursor	3,25	0,002
ENSG00000129277	CCL4	Small inducible cytokine A4 precursor	5,87	0,001

No genes were differentially expressed in the controls at T = 0 vs. T = 1.

For the T = 0 vs. T = 4 experiments, 10 arrays were hybridised with LPS-stimulated samples and 4 arrays with control samples. On average 26% of spots passed the stringent quality control criteria. In total 28 genes were identified as being differentially expressed in monocytes following LPS stimulation, of which 3 were down regulated and 25 up regulated (see Table 2 and Figure 3). The fold changes ranged from 2.0 to 5.7. Interestingly, *AKIP *was found to be differentially expressed at both one and four hours after LPS infusion.

**Table 2:** Genes differentially expressed four hours after LPS infusion

Ensembl ID	HUGO geneID	Full name	foldchange	p-value
ENSG00000168383	HLA-DPB1	HLA class II histocompatibility antigen, DP(W4) beta chain precursor	0,44	4,2E-05
ENSG00000152518	ZFP36L2	Butyrate response factor 2	0,47	6,7E-05
ENSG00000158050	DUSP2	Dual specificity phosphatase 2	0,47	9,3E-04
ENSG00000148218	ALAD	Delta-aminolevulinic acid dehydratase	2,04	8,2E-04
ENSG00000152684	PELO	Pelota homolog	2,08	6,0 E-04
ENSG00000163220	S100A9	Calgranulin B	2,09	3,8 E-03
ENSG00000088986	DNCL1	Dynein light chain 1, cytoplasmic	2,15	8,2 E-04
ENSG00000134802	SLC43A3	Solute carrier family 43, member 3	2,17	3,1 E-03
ENSG00000163754	GYG	Glycogenin-1	2,21	1,1 E-03
ENSG00000084733	RAB10	Ras-related protein Rab-10	2,23	9,0 E-04
ENSG00000137312	FLOT1	Flotillin-1	2,23	2,6 E-03
ENSG00000023330	ALAS1	5-aminolevulinate synthase, nonspecific, mitochondrial precursor	2,24	1,6 E-03
ENSG00000015475	BID	BH3 interacting domain death agonist	2,27	2,6 E-03
ENSG00000123405	NFE2	Transcription factor NF-E2 45 kDa subunit	2,27	2,9 E-03
ENSG00000096238	CLIC1	Chloride intracellular channel protein 1	2,29	4,8 E-04
ENSG00000168439	STIP1	Stress-induced-phosphoprotein 1	2,29	3,1 E-03
ENSG00000101310	SEC23B	Protein transport protein Sec23B	2,32	6,0 E-04
ENSG00000106211	HSPB1	Heat-shock protein beta-1	2,33	2,1 E-03
ENSG00000102265	TIMP1	Metalloproteinase inhibitor 1 precursor	2,33	3,1 E-03
ENSG00000170458	CD14	Monocyte differentiation antigen CD14 precursor	2,40	4,8 E-04
ENSG00000155926	SLA	SRC-like-adapter	2,41	1,9 E-04
ENSG00000109971	HSPA8	Heat shock cognate 70 kDa protein	2,45	4,6 E-04
ENSG00000038427	VCAN	Versican core protein precursor	2,45	8,5 E-06
ENSG00000106803	SC61B	Protein transport protein Sec61 beta subunit	2,46	8,9 E-05
ENSG00000137462	TLR2	Toll-like receptor 2 precursor	2,77	2,5 E-04
ENSG00000156127	BATF	ATF-like basic leucine zipper transcriptional factor B-ATF	3,13	2,3 E-04
ENSG00000171860	C3aR1	C3a anaphylatoxin chemotactic receptor	4,65	1,0 E-03
ENSG00000175756	AKIP	Aurora kinase A-interacting protein	5,68	8,7 E-04

No genes were differentially expressed in the controls at T = 0 vs. T = 4.

We did not identify any genes that were significantly differentially expressed in the control experiments at either T = 1 or T = 4 confirming that the differential expression observed, whilst limited, is associated with LPS infusion.

#### Microarray data validation

Using quantitative RT-PCR, we were able to confirm the differential expression of 7 of 8 genes tested (Figure 3). Validation was performed on RNA from individuals who had not been part of the subset analysed by microarray, thus highlighting that the changes observed are universal. Absolute fold changes varied between the micro-array and Taqman results, however in all cases, except for *AKIP *(both at T = 1 and T = 4), the direction of differential expression was conserved.

### In Vitro Experiments

In the *in vitro *experiments a total 1127 genes were differentially expressed due to stimulation with LPS (ArrayExpress accession number E-TABM-483). Six of these genes were also differentially expressed in our *in vivo *study. For all results see additional file 4, supplementary table 3.

## Discussion

We applied a stepped approach in our study to provide evidence that endotoxin exposure *in vivo *results in a proatherogenic phenotype in circulating monocytes. First, we proved that the LPS infusion model we applied results in both clinical symptoms and increased IL-6, a marker for inflammation associated with TLR4 signaling (17), with IL-6 levels peaking at three hours post LPS infusion. Second, circulating monocytes were profiled on cDNA arrays pre and post LPS infusion. We generated a list of 39 genes differentially expressed at one or four hours post LPS infusion. A lack of overlap between genes at both time points was observed, with only *AKIP *being differentially expressed at both time points. This observation is likely due to the sequential activation of pathways after the LPS stimulus. In addition, this might reflect different populations of monocytes contributing to the transcriptome, since only 45% of monocytes remain in circulation one hour after LPS stimulation. Of these genes, 7 of 8 randomly selected candidates were confirmed by RT-PCR. Finally, comparisons with our *in vitro *data identified 6 overlapping genes (*BATF, BID, C3aR1, IL1RN, SEC61B *and *SLC43A3*). Since *C3aR1 *has been previously associated with inflammation and atherosclerosis this might be one of the interesting candidates in our experiments to link both disorders. We will focus our discussion on the genes which were differentially expressed in both our *in vivo *and *in vitro *experiments.

The *C3aR1 *gene is located on chromosome 12. The gene product is a highly ligand-specific membrane receptor belonging to the family of the seven transmembrane domain G-protein-coupled receptors. *C3aR1 *is expressed in monocytes, macrophages and endothelial cells. Binding of C3 to C3aR1 induces a wide rang of inflammatory and immune effects [11]. There are multiple lines of evidence for the role of *C3aR1 *in atherosclerosis.

At gene expression level, in samples of patients with advanced peripheral artery disease (PAD) the gene was expressed at a five times higher level in advanced compared to intermediate atherosclerotic lesions [12]. At protein level, advanced human coronary atherosclerosis plaques express *C3aR1 *in contrast to normal coronary intima [13]. In addition, signaling via *C3aR1 *promotes plaque instability and can thus result in clinical sequelae of acute coronary syndromes [13].

The direct causal role of *C3aR1 *is provided by the fact that pertubaration of this gene in knock out mice models result in significant decrease in atherosclerotic lesion size [14].

Putting the evidence together, we speculate the following sequence of events. We realize that several pathways will co-exist and this is merely one of them. Low grade inflammation due to endotoxinemia results in the higher expression of *C3aR1 in vivo*. Increased *C3aR1 *expression in turn results in a proatherogenic monocyte phenotype. Firstly, this results in increased atherosclerotic lesion size. Secondly, it results in increased plaque instability. Ultimately all these effect might result in more clinical CVD events.

The up regulation of *IL1RN *and *BATF*, in contrast, does not support our hypothesis of the circulating monocyte with a proatherogenic phenotype.

*IL1RN *is a cytokine gene located on chromosome 2. It is a negative regulator of IL-1 signalling and plays a role as an anti-inflammatory cytokine in acute and chronic- inflammation of the vascular wall. It is expressed by macrophages, endothelial cells and smooth muscle cells. Endogenous IL-Ra suppresses atherosclerosis in humans. Decreased expression and not increased expression has been associated with atherosclerotic plaques in mice [15]. The increased expression of IL1RN might be an internal protective mechanism to downlplay the effects of systemic inflammation due to the LPS stimulus.

*BATF *is a nuclear basic leucine zipper protein and a member of the AP-1 family of transcription factors located on chromosome 14. It directly regulates key components of the formation and function of follicular helper T cells and antibody class switching in B cells [16]. In B-cells the expression of BATF is also induced by LPS and IL-6 [16]. Expression of *BATF *in rat fibroblast suppressed the production of Metalloproteinase (MMP)-2 and MMP-9. MMPs are key players in atherothrombosis due to their extracellular matrix remodelling properties and their functional effects on cells involved in atherogenesis and atherosclerotic complications [17].

The role of *BID, SEC61B, SLC43A3 *in atherosclerosis is not yet established.

*BID *is located on chromosome 22q and is a critical mediator of inflammation and innate immunity [18]. Mechanistically, BID interacts with NOD1, NOD2 and the IκB kinase (IKK) complex, impacting NF-κB and extracellular signal-regulated kinase (ERK) signalling [18]. Targeting BID by small molecules has been proposed as a way to treat immune-mediated inflammatory disease including inflammatory bowel disease.

*SEC61B *is located on chromosome 9q22. In the Endoplasmatic Recticulum (ER) membrane, the heterotrimeric Sec61 complex comprises three transmembrane subunits (Sec61α, Sec61β, and Sec61γ in mammals) and forms protein-conducting channels, collectively termed a translocon. Sec61α is known to be stabilized by Sec61γ and mainly responsible for the translocation activity in the ER. In contrast to the other two subunits, Sec61β can be stable on its own, and its function is not as well defined. It is known that Sec61β in the inner nucleus membrane (INM) is required for the release of epidermal growth factor from the INM to the nucleus [19].

*SLC43A3 *is located on chromosome 11 and is known to be highly expressed in macrophages. It is thought to function as a transporter of metabolites and nutrients that are necessary during developmental events, such as organogenesis [20]. It is part of the specific expression pattern of the micro vascular endothelium and has been proposed as putative drug target to pathological angiogenesis [21].

Our study has several limitations. The experiments were conducted in a small number of individuals. However the results are supported by the fact that the selected candidate genes were validated by RT-PCR. In addition, several genes have been previously described in the literature as being differentially expressed in response to endotoxin exposure [22,23]. In our experiment we determined the effect of LPS on gene expression in circulating monocytes. However, a substantial part of the monocytes migrated into the vessel wall, reflected by a fall in monocyte count in the peripheral blood after LPS infusion. Therefore it begs questioning how representative these circulating monocytes are for the entire population of these cells [24]. In this respect, the overlap in genes differentially expressed in our *in vivo *study compared to previous *in vitro *LPS challenges, lends further support to the validity of the identified candidate genes in circulating monocytes. However, the low level of homology between *in vivo *and *in vitro *transcriptomes makes us question the value of studying expression profiles of circulating cells as (prognostic) markers for disease states. The poor overlap between *in vivo *and *in vitro *experiments could be due to either the intrinsic differences between the two systems or due to the small sample sizes in both studies.

Another limitation is that the procedure of monocyte isolation is time consuming and might thus effect transcription and stability. To minimize these potential effects, for all *in vivo *and *in vitro *experiments the same optimized protocol was used by the same analyst. Furthermore genes differentially expressed due to handling would have showed up in the placebo experiments. No genes were differentially expressed in these experiments.

Finally, in comparison to *in vitro *experiments only a small number of genes were differentially expressed. This is in part due to the stringent quality control criteria we applied during the analysis, the limited coverage of the microarrays and the fact that TLR4 signaling is modulated in vivo by a number of specific pathways.

## Conclusion

Endotoxin exposure in *in vivo *to healthy individuals identified several candidate genes through which systemic inflammation can result in accelerated atherosclerosis. Out of these candidates, *C3aR1*, might be a promising target, solely based on the existing literature. Our results in combination with previous reports support the possible role of anaphylatoxins and the complement system as potential target for treatment of acute sequels of atherosclerosis through plaque stabilization.

## Conflict of interest

The authors declare that they have no competing interests.

## Authors' contributions

WHO, NW and JJZ designed the study. SS, RF, RB and MN designed and carried out the experiments. AF performed the statistical analysis. CL and NW supervised the experiments. SS, NW and MDT wrote the manuscript. SS, RF, AG, NW and SM revised the manuscript. All authors have read and approved the final manuscript.

## Pre-publication history

The pre-publication history for this paper can be accessed here:

http://www.biomedcentral.com/1755-8794/4/64/prepub

## Supplementary Material

Additional file 1 **Supplementary Methods**. Full methods of the *in vitro *experiments. Click here for file

Additional file 2 **LPS Supplementary Table 1**. RNA yield and quality data for all samples and those included in the study. Click here for file

Additional file 3 **LPS Supplementary Table 2**. cDNA quantity, quality, labeling and amplification data for all samples and those included in the study. Click here for file

Additional file 4 **Supplementary Table 3 Ctrl vs LPS *in vitro***. Full results of the *in vitro *experiments. Click here for file
