# Supplementary material for: Identification of candidate genes linking systemic inflammation to atherosclerosis; results of a human in vivo LPS infusion study
Source: BMC Med Genomics. 2011 Aug 10;4:64. doi: 10.1186/1755-8794-4-64 (PMC3174875; doi:10.1186/1755-8794-4-64)
Supplement: Additional file 1 — Supplementary Methods. Full methods of the in vitro experiments. [file 1755-8794-4-64-S1.DOC]

**Supplementary Methods**

*Whole blood Endotoxin incubation*

Citrate anticoagulated whole blood samples (n=8) were split into 2 aliquots and incubated for 4 h at 37 °C and 5% CO_2_ with either pyrogen free carrier or 5 ng/mL LPS from *E. coli* serotype O113:H10:K (NIBSC, Potters Bar, UK).

*Monocyte mRNA preparation*

After 4 h, mononuclear cells (MNCs) were purified by Histopaque 1077 (Sigma, Poole, UK) gradient centrifugation followed by positive selection of CD14^+^ monocytes using magnetic cell sorting (EasySep Human CD14 Selection Kit, Stem Cell Technologies, London, UK) following the manufacturer’s instructions. Monocyte purity further confirmed by by flow cytometry using 10 μl each of FITC-anti-CD45 and PE-anti-CD14 (Dako Ltd, Ely, UK) and real-time PCR using the lineage specific markers as described above. RNA was extracted from purified monocytes using Trizol according to manufacturer’s instructions (Invitrogen, Paisley, UK). Quantification of the resulting RNA was carried out using a NanoDrop spectrophotometer (Labtech International, Ringmer, UK) and quality, measured as the 18S/28S rRNA ratio, was assessed on an Agilent BioAnalyzer (Agilent Technologies, Stockport, UK).

*Monocyte cRNA preparation*

From these samples 100 ng total RNA was amplified using the Illumina TotalPrep RNA amplification kit following the manufacturer’s instructions (Applied Biosystems) with *in vitro* transcription for 16 h at 37 °C.

*Microarray processing*

Amplified cRNA (1.5 μg in 20 μl) was hybridised onto Illumina HumanWG-6 v1 arrays. Hybridised cRNA was detected using streptavidin-Cy3. Arrays were scanned on an Illumina BeadArray Reader and the image data extracted using the BeadStudio software package (all from Illumina, Saffron Walden, UK). All data were deposited into ArrayExpress accession number E-TABM-483.

*Data analysis*

Statistical analysis of microarray data was carried out using R statistical package (http://www.r-project.org). Raw data was normalised using quantile normalizationand the data filtered to include only features with detection scores above background [11] . Paired analysis using Student’s t-test was carried out for Ctrl *vs.* LPS. A fold change and a p-value with false discovery rate (FDR2d) correction for multiple testing were computed for each feature [11]. To identify significant, differentially expressed genes, the output was filtered on FDR2d corrected p-values ≤0.05 and fold changes ≥2. Probes were mapped to the corresponding genes using the Ensembl BioMart tool (http://www.biomart.org).

*Microarray results validation*

Expression changes of randomly selected transcripts were validated by real-time PCR using the following probes: *IRF7* (Hs00185375_m1), *NFKBI* (Hs00765730_m1), *PPARG* (Hs00234592_m1), *CD300c* (Hs00198564_m1), *CLEC12A* (Hs00370620_m1), *CLECSF5* (Hs00183780_m1), *Gi24* (Hs00735289_m1), *MERTK* (Hs00179024_m1), *OLR1* (Hs00234028_m1), *THBD* (Hs00264920_s1), and *ADFP* (Hs00605340_m1) (all from Applied Biosystems). All RT-PCR assays were carried out in triplicate on 0.5 ng RNA and following manufacturer’s instructions.
