# Supplementary material for: Identification of candidate genes linking systemic inflammation to atherosclerosis; results of a human in vivo LPS infusion study
Source: BMC Med Genomics. 2011 Aug 10;4:64. doi: 10.1186/1755-8794-4-64 (PMC3174875; doi:10.1186/1755-8794-4-64)
Supplement: Additional file 2 — LPS Supplementary Table 1. RNA yield and quality data for all samples and those included in the study. [file 1755-8794-4-64-S2.DOC]

**Supplementary** Table 1: Sample details

|  |  | **All samples** |  | **Selected samples** |  | **Selected samples** |  |
| --- | --- | --- | --- | --- | --- | --- | --- |
|  |  |  |  | **T=0 vs T=1 comparison** |  | **T=0 vs T=4 comparison** |  |
|  |  | *Purity (%)* | *RNA yield (ng)* | *Purity (%)* | *RNA yield (ng)* | *Purity (%)* | *RNA yield (ng)* |
|  |  |  |  |  |  |  |  |
| **LPS** | T=0 | 85.45 ± 15.18 | 574.3 ± 519.0 | 89 ± 5.3 | 608.1 ± 648.6 | 93.2 ± 2.2 | 692.3 ± 371 |
|  | T=1 | 69.89 ± 21.6 | 221 ± 313.7 | 80.6 ± 6.6 | 205.7 ± 184.9 |  |  |
|  | T=4 | 92.64 ± 3.931 | 1013 ± 539.5 |  |  | 92.8 ± 3.0 | 907.4 ± 186.3 |
|  |  |  |  |  |  |  |  |
| **Control** | T=0 | 87 ± 8.5 | 928.7 ± 589.8 | 91 ± 3.4 | 721.4 ± 420.9 | 92 ± 4.2 | 566 ± 240.4 |
|  | T=1 | 88 ± 8.0 | 518.4 ± 300.6 | 94.5 ± 1.7 | 884.3 ± 441.1 |  |  |
|  | T=4 | 94 ± 2.5 | 1011± 475.9 |  |  | 92.5 ± 3.5 | 704.8 ± 148.2 |
|  |  |  |  |  |  |  |  |

Values are represented as mean ± SD
