# Supplementary material for: Identification of candidate genes linking systemic inflammation to atherosclerosis; results of a human in vivo LPS infusion study
Source: BMC Med Genomics. 2011 Aug 10;4:64. doi: 10.1186/1755-8794-4-64 (PMC3174875; doi:10.1186/1755-8794-4-64)
Supplement: Additional file 3 — LPS Supplementary Table 2. cDNA quantity, quality, labeling and amplification data for all samples and those included in the study. [file 1755-8794-4-64-S3.DOC]

**Supplementary** Table 2: Details samples used for microarray experiments

|  |  |  | **cDNA quantity and quality** |  | **Labelling and amplification** |  |
| --- | --- | --- | --- | --- | --- | --- |
| Comparison | Infusion |  | *yield* | *A260/A280* | *Average dye incorporation* | *Fold increase cDNA* |
| T=0 vs. T=1 | LPS |  | 5.7 ± 1.7 | 1.89 ± 0.11 | 4.73 ± 2.66 | 44 ± 17 |
| T=0 vs. T=1 | NaCl |  | 5.7 ± 1.6 | 1.87 ± 0.03 | 5.73 ± 1.56 | 45 ± 16 |
|  |  |  |  |  |  |  |
| T=0 vs. T=1 | LPS |  | 3.9 ± 1.8 | 1.84 ± 0.03 | 4.39 ± 2.66 | 47 ± 18 |
| T=0 vs. T=4 | NaCl |  | 4.8 ± 2.5 | 1.86 ± 0.01 | 4.67 ± 2.15 | 46 ± 15 |
|  |  |  |  |  |  |  |
| Values are represented as mean ± SD | | | |  |  |  |
